# Karyotyping and Distribution Patterns of Endemic Chinese Lilies: Insights Into Their Conservation Under Climate Change

**DOI:** 10.1002/ece3.72824

**Published:** 2026-01-11

**Authors:** Tengfei Gui, Mingyue Lin, Zhiming Li, Deli Peng, Yuan Huang, Wenguang Sun

**Affiliations:** ^1^ School of Life Science Yunnan Normal University Kunming China; ^2^ Engineering Research Center for Valorization of Unique Bio‐Resources in Yunnan, Ministry of Education, School of Life Sciences Yunnan Normal University Kunming China

**Keywords:** Alpine environments correlation analysis, climate change, karyotype indices, *Lilium lophophorum*, *Lilium nanum*, MaxEnt

## Abstract

This study integrates cytogenetic and ecological analyses of two endemic Chinese alpine lilies, *Lilium lophophorum* (2*n* = 24) and *L. nanum* (2*n* = 48), to establish a foundational understanding of their chromosomal diversity and distribution patterns. We document substantial intraspecific karyotypic variation in diploid *L. lophophorum*, with preliminary associations to altitude, and provide the first chromosomal characterization of tetraploid *L. nanum* . Ecological niche modeling under future climate scenarios predicts upward range shifts for both species, with the tetraploid exhibiting greater potential for habitat expansion. The distribution patterns, combined with the dwarf phenotype of *L. nanum* , support the hypothesis that polyploidy may enhance resilience in extreme high‐altitude environments. However, the limited sample size warrants interpreting these results as hypothesis‐generating rather than demonstrating adaptive superiority. This work offers a theoretical framework for further study, highlighting the need for broader taxonomic and geographic sampling and genomic analyses to test the link between polyploidy and environmental adaptability. These insights also inform conservation planning by emphasizing the protection of high‐altitude refugia under climate change.

## Graphical abstract

This study integrates cytogenetic and ecological analyses of two endemic Chinese alpine lilies, *Lilium lophophorum* (2*n* = 24) and *L. nanum* (2*n* = 48), to establish a foundational understanding of their chromosomal diversity and distribution patterns. We document substantial intraspecific karyotypic variation in diploid *L. lophophorum*, with preliminary associations to altitude, and provide the first chromosomal characterization of tetraploid *L. nanum* . Ecological niche modeling under future climate scenarios predicts upward range shifts for both species, with the tetraploid exhibiting greater potential for habitat expansion. The distribution patterns, combined with the dwarf phenotype of *L. nanum* , supports the hypothesis that polyploidy may enhance resilience in extreme high‐altitude environments. However, the limited sample size warrants interpreting these results as hypothesis‐generating rather than demonstrating adaptive superiority. This work offers a theoretical framework for further study, highlighting the need for broader taxonomic and geographic sampling and genomic analyses to test the link between polyploidy and environmental adaptability. These insights also inform conservation planning by emphasizing the protection of high‐altitude refugia under climate change.

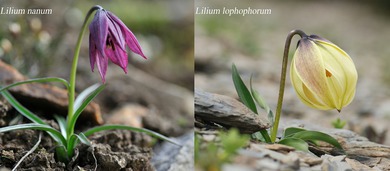

## 1 Introduction

The Qinghai‐Tibet Plateau (QTP) represents one of the world's most extreme environments, characterized by high elevation, intense UV radiation, and large diurnal temperature fluctuations (Zhang, Li, et al. 2021; Zhang, Liu, et al. 2021). These conditions have driven the evolution of diverse adaptive strategies in the region's flora, which includes roughly 3000 plant species representing nearly 30% of the world's alpine plants (Nie et al. 2004; Yang et al. 2019; Liu et al. 2023). Adaptive divergence in QTP plants is frequently attributed to environmental heterogeneity across elevation gradients. Altitude integrates shifts in temperature, humidity, atmospheric pressure, and UV radiation, factors that directly shape plant physiology, metabolism, and genetic structure (Long and Hutchin 1991; Tranquillini 2012; Magaña et al. 2019).

Studies show that even small altitudinal differences can significantly influence genetic diversity and reproductive strategies in plants. For example, a study on *Juniperus* in the Hengduan Mountains found that an elevation change of only 150 m influenced its genetic diversity (Ju et al. 2022). Research on woody plants in the Changbai Mountains reported decreasing tree species diversity with increasing elevation (S. J. Li 2022), and studies on *Elymus* species showed that variation in reproductive traits was most pronounced at high altitudes (C. Zhao 2017). Together, these findings indicate that environmental heterogeneity along elevation gradients is a major driver of phenotypic and genetic variation within species.

Despite significant progress in understanding ecological adaptation in QTP plants, the genomic and chromosomal bases of these adaptations remain insufficiently characterized. Strong selective pressures have produced specialized traits expressed not only phenotypically but also through genomic and chromosomal variation (N. Zhao 2021). The QTP flora shows substantial chromosomal diversity, including high levels of polyploidy across multiple families and genera (Zhang, Peng, et al. 2021). Such chromosomal variation is a major source of genetic diversity and is widely considered a key driver of speciation and ecological adaptation, supporting niche differentiation and tolerance to environmental stress (Wang et al. 2001). Integrating climate and species distribution data, Rice et al. (2019) further demonstrated that the frequency of polyploidy generally increases from low to high latitudes across 26,599 plant species in 1287 genera (Rice et al. 2019).

China's flora includes approximately 20,000 native plant species, with the genus *Lilium* notable for its ornamental and ecological importance. Of the 110–115 global *Lilium* species, China hosts 55 species and 18 varieties. The region's diverse climates and topographies have shaped substantial morphological diversity and environmental resilience (Zhao et al. 1994; Wu et al. 2006; H. J. Li 2007). Two endemic alpine species, the diploid *Lilium lophophorum* (2*n* = 24) and tetraploid *L. nanum* (2*n* = 48), provide an ideal model for studying high‐altitude adaptation. *L. lophophorum* has smooth stems, lanceolate leaves, and yellow flowers with purple‐red spots, and typically occurs at 2700–4250 m. In contrast, the dwarf *L. nanum* bears pale purple or pink flowers and occupies colder, nutrient‐poor habitats at 3500–4500 m (Liang and Tamura 2000; Sun et al. 2014). Their distinct altitudinal ranges suggest differential environmental adaptation, offering a natural system for investigating the potential role of ploidy (Zhang et al. 2023).

However, the relationship between chromosomal structure, particularly ploidy level, and environmental adaptation in *Lilium* remains poorly understood. To address this gap, we apply the Maximum Entropy (MaxEnt) model, a widely used tool for predicting species distributions that has shown strong performance in ecological studies (Zhang et al. 2017). For example, MaxEnt has been used to identify suitable cultivation areas for jujube in Xinjiang (Zhang, Lu, et al. 2020; Zhang, Zhao, and Wang 2020) and to project the distribution of *Populus euphratica* under future climate scenarios (Zhang, Lu, et al. 2020; Zhang, Zhao, and Wang 2020). Species distribution models (SDMs) such as MaxEnt integrate geographic occurrence data and environmental variables to link species distributions to environmental factors, making them well suited for examining adaptive evolution in alpine environments (Zhu et al. 2013).

This study uses an integrative approach to test the hypothesis that polyploidy enhances plant adaptability to extreme environments. We first analyze intraspecific karyotypic diversity in diploid *L. lophophorum* across multiple populations to establish a baseline of chromosomal variation. Building on this cytogenetic foundation, we combine chromosomal data with MaxEnt ecological niche modeling. By comparing distribution patterns and niche differentiation between tetraploid *L. nanum* and its diploid relative under current and future climate scenarios across the QTP, we aim to provide empirical evidence for a polyploidy‐driven adaptive advantage. Integrating chromosomal analysis with ecological modeling offers a novel framework for assessing how polyploidy may influence species' responses to climate change. This work seeks to clarify the mechanistic role of chromosomal variation in adaptation to extreme environments and to inform conservation strategies for alpine species under global climate change.

## 2 Materials and Methods

### 2.1 Plant Materials and Collection

**FIGURE 1:**
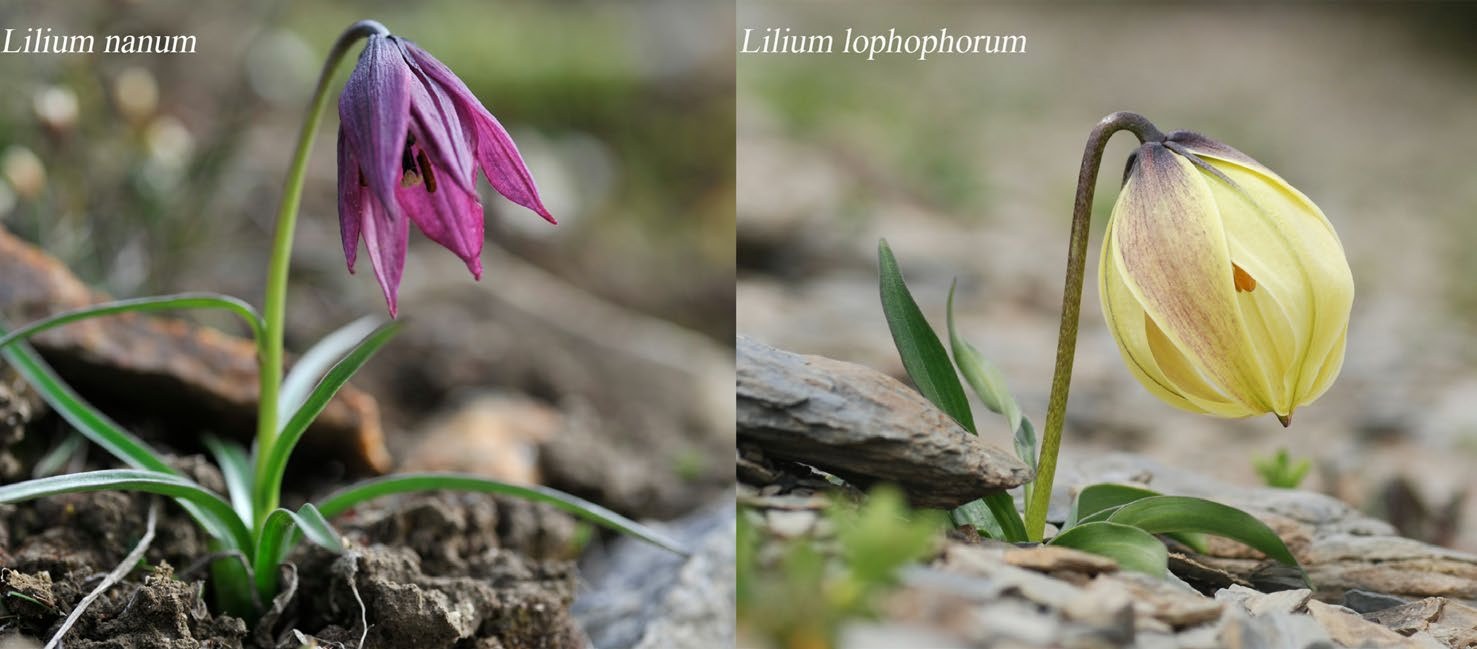
Morphological comparison of the two studied *Lilium* species in their natural habitats. Left: *Lilium nanum* with a fully opened, deep purple, nodding flower, narrow linear leaves near the ground, and a slender green stem, growing in coarse dark brown soil. Right: *Lilium lophophorum* with a bright yellowish‐green, partially opened, tightly wrapped flower, a more robust stem, and broader ascending leaves, occurring in a brighter microhabitat among light‐colored rocks. Both photographs were taken in the wild.

**FIGURE 2:**
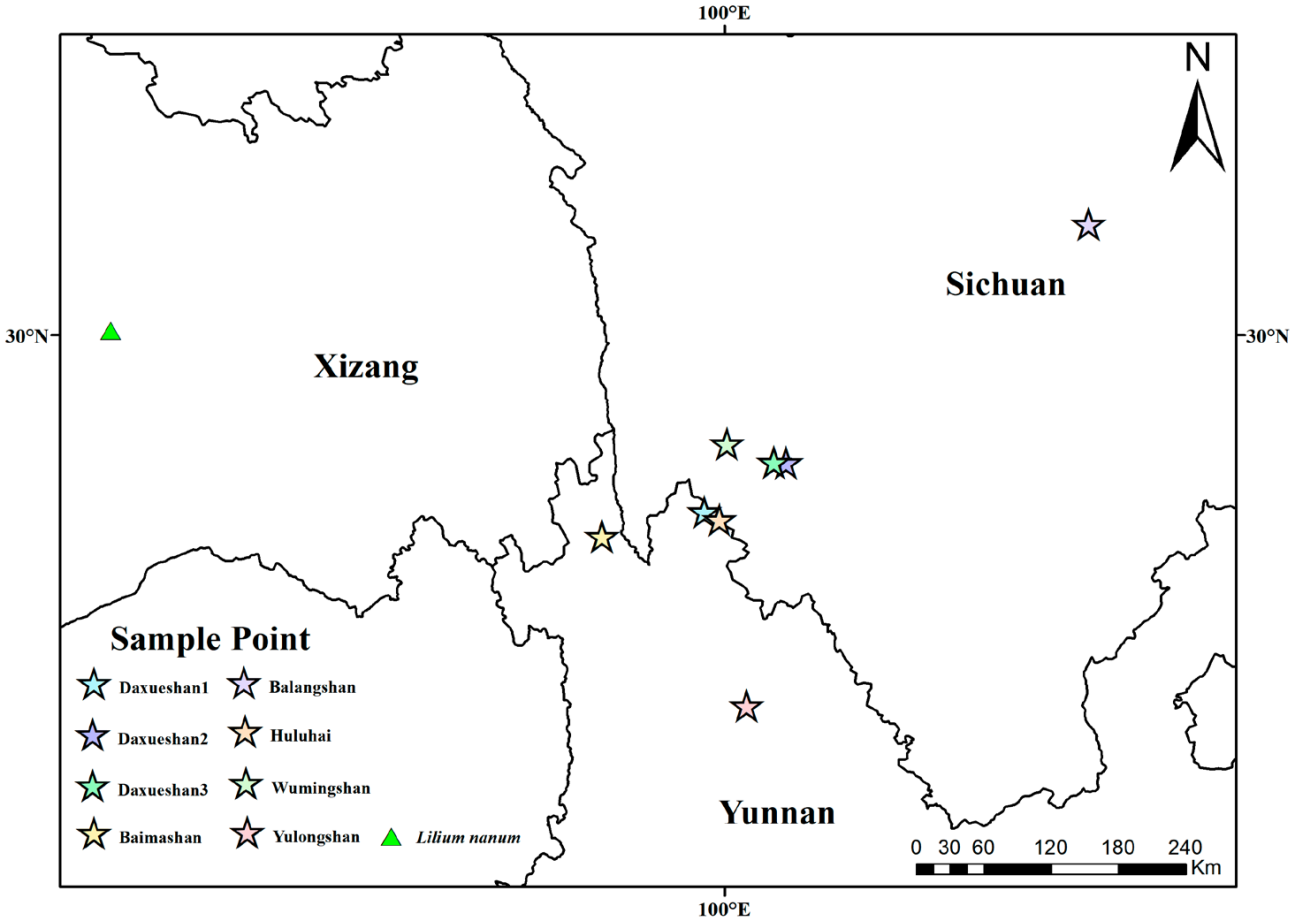
Sampling location map (triangles denote *Lilium nanum*; pentagons denote *Lilium lophophorum*).

Plant materials were collected from multiple locations across the QTP. Because of the region's difficult terrain, the sampling strategy targeted one individual per population to maximize spatial coverage. Voucher specimens are deposited in the Herbarium of the Kunming Institute of Botany, Chinese Academy of Sciences (KUN), and slide specimens are housed in the Plant Cytology Laboratory at Yunnan Normal University. Representative plant material is shown in Figure 1, and detailed sampling locations are provided in Table 1 and Figure 2.

**TABLE 1:** Specimen sampling locations.

Species	Collection site	District (Designation)	Coordinates	Altitude (m)	Voucher (KUN)
*L. lophophorum*	Xiangcheng, Sichuan	Daxue Mountain (DXS1)	E 99°50′21.13″; N 28°35′3.83″	4509	PSH‐134
*L. lophophorum*	Shangri‐La, Yunnan	Daxue Mountain (DXS2)	E 99°88′97.98″; N 28°58′44.76″	4335	YNNU‐19‐124
*L. lophophorum*	Shangri‐La, Yunnan	Daxue Mountain (DXS3)	E 99°83′57.23″; N 28°58′57.26″	4280	YNNU‐19‐146
*L. lophophorum*	Lijiang, Yunnan	Yulong Snow Mountain (YLS)	E 100°10′45.68″; N 27°01′54.66″	3924	SunWG‐0286
*L. lophophorum*	Shangri‐La, Yunnan	Geza Township Huluhai (HLH)	E99°57′45.691”; N 28°31′16.802″	4693	SunWG‐0320
*L. lophophorum*	Xiangcheng, Sichuan	Wuming Mountain (WMS)	E 100°1′19.79″; N 29°7′45.34″	4525	SunWG‐0220
*L. lophophorum*	Shangri‐La, Yunnan	Baimashan Snow Mountain (BMS)	E 99°1′17.68″; N 28°23′15.63″	4471	PengDL‐370
*L. lophophorum*	Wenchuang, Sichuan	Balang Mountain (BLS)	E 102°55′14.61”; N 30°53′27.23″	3852	PengDL‐714
*L. nanum*	Linzhi, Xizang	Sejila Mountain (SJL)	E 94°65′14.06″; N 29°61′62.21″	4644	PengDL‐153

### 2.2 Chromosome Preparation and Karyotypic Analysis

#### 2.2.1 Chromosome Preparation

*Lilium* root tips were treated with either 0.003 mol/L 8‐hydroxyquinoline or 0.1% colchicine at 20°C–21°C for 4–5 h. They were then fixed in Carnoy's solution (ethanol: acetic acid = 3:1) at 4°C for 4–12 h, softened in 1 N HCl at 60°C for 7 min, stained with Carbol fuchsin for 6–9 h, and finally smeared onto glass slides.

#### 2.2.2 Karyotype Characterization

Images of six metaphase cells with clear, well‐dispersed chromosomes were captured. Chromosomal arm lengths were measured using Karyotype V2.0 software (Altınordu et al. 2016). Centromeric positions were determined following Levan et al. (Levan et al. 2009). Karyomorphological classification of mitotic interphase nuclei and prophase chromosomes followed Tanaka (Tanaka 1971), and karyotype asymmetry was assessed according to Stebbins (Stebbins 1971).

#### 2.2.3 Statistical Analysis of Karyotypic Data

A comprehensive pairing analysis was conducted for the six cells. Relationships between karyotypic parameters and environmental factors (including annual mean temperature, annual precipitation, slope, aspect, and altitude extracted from collection coordinates) were examined using Pearson correlation coefficients and Principal Component Analysis (PCA) (Basu 2023). Analyses were performed with Origin 2022 and SPSS 26.

### 2.3 Species Distribution Modeling

#### 2.3.1 Species Occurrence Data

Distribution data for diploid *L. lophophorum* and tetraploid *L. nanum* were obtained from the Chinese Virtual Herbarium (CVH), the Global Biodiversity Information Facility (GBIF), and the National Specimen Information Infrastructure (NSII). The raw data were cleaned in R to remove duplicate records and points heavily influenced by human activity, yielding 83 valid records for *L. lophophorum* and 81 for *L. nanum* for subsequent analyses.

#### 2.3.2 Environmental Variables

Initial environmental variables included 19 bioclimatic factors from WorldClim, along with elevation, slope, and aspect (the latter two derived from a Digital Elevation Model). A correlation analysis was conducted, and variables with correlation coefficients (r) ≥ 0.8 were considered highly intercorrelated (Ning et al. 2018; Yang et al. 2024). Final variable sets for each species were selected based on their contribution rates and lower intercorrelation (Chen et al. 2024).

#### 2.3.3 Model Implementation and Evaluation

Species distribution modeling was conducted using the MaxEnt algorithm. The model was run in Auto‐feature mode with a regularization multiplier of 1, ten subsample replicates, and a 75%/25% split of occurrence data for training and testing. Model performance was assessed using the Area Under the Receiver Operating Characteristic Curve (AUC) (Wang et al. 2007), with AUC values > 0.9 indicating excellent predictive accuracy (Gao et al. 2018).

#### 2.3.4 Habitat Suitability Mapping

Model outputs were processed in ArcGIS 10.4. The natural breaks classification method was used to categorize areas into suitable and unsuitable habitats. Potential distributional changes for both species were inferred from the resulting raster data.

## 3 Results

**FIGURE 3:**
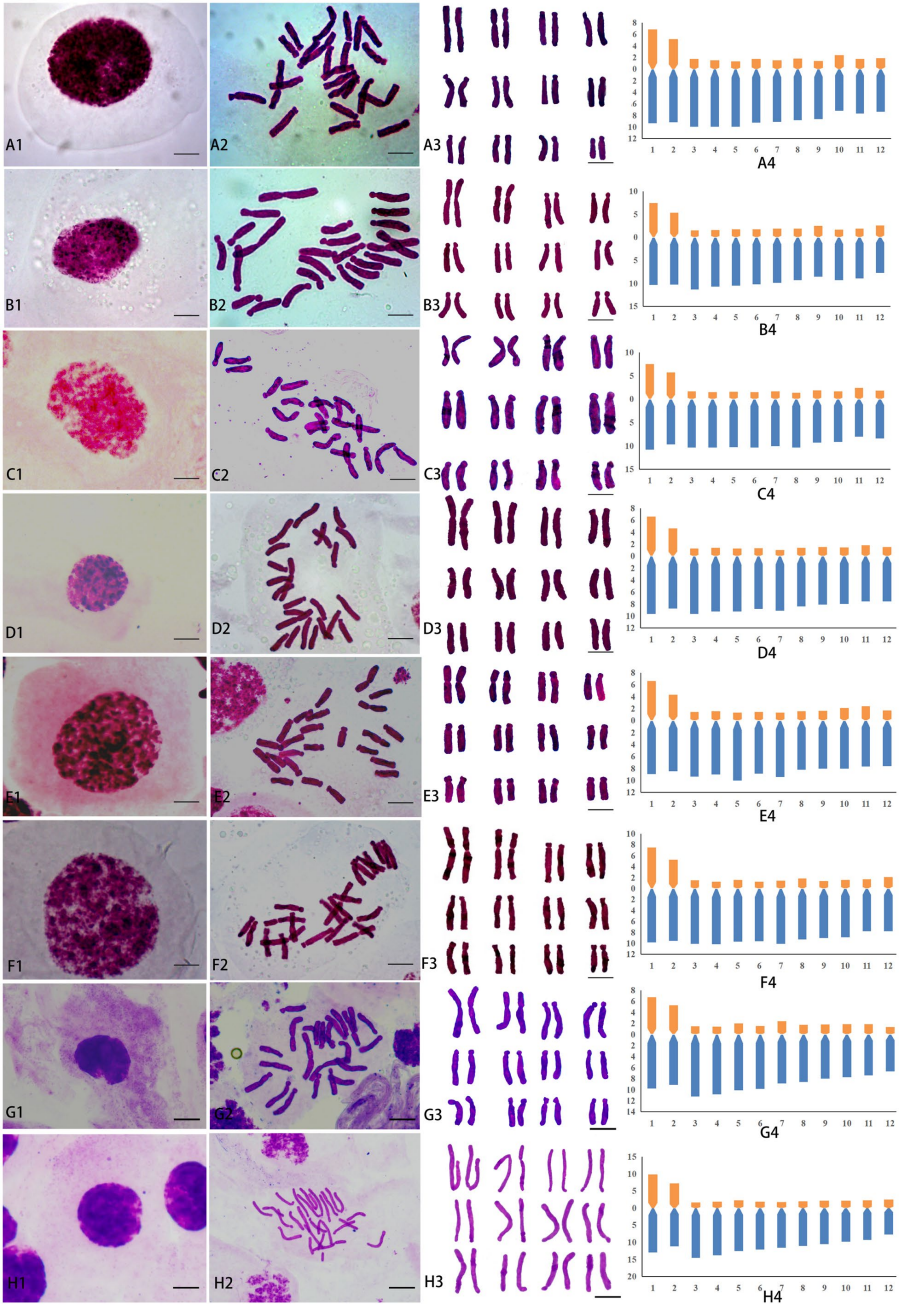
Mitotic cytograms and karyotype patterns of eight *Lilium lophophorum* populations. A1‐H1: Mitotic interphase. A2‐H2: Metaphase. A3‐H3: Chromosome segmentation and analysis. A4‐H4: Ideograms. A1‐A4: DXS1. B1‐B4: DXS2. C1‐C4: DXS3. D1‐D4: YLS. E1‐E4: HLH. F1‐F4: WMS. G1‐G4: BMS. H1‐H4: BLS. Red indicates the short arm and blue indicates the long arm. Y‐axis: Chromosome length (μm). X‐axis: Chromosome number. Scale bar = 5 μm.

**FIGURE 4:**
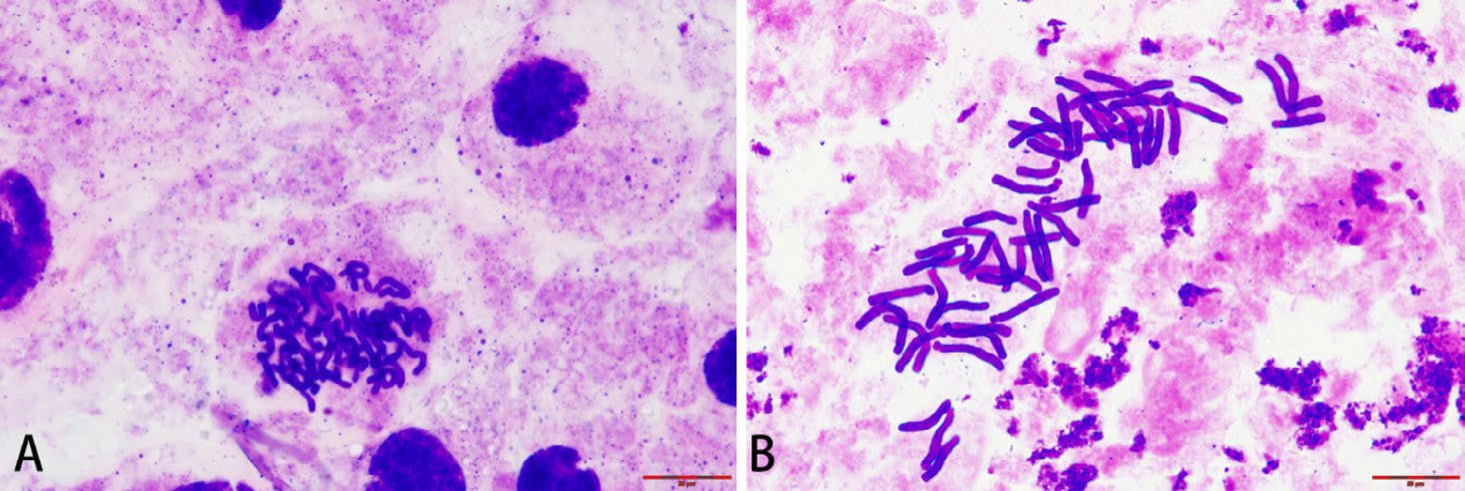
Mitotic cytograms of *Lilium nanum* reported here for the first time. (A) Mitotic interphase. (B) Metaphase (2*n* = 4*x* = 48). Scale bar = 20 μm.

**FIGURE 5:**
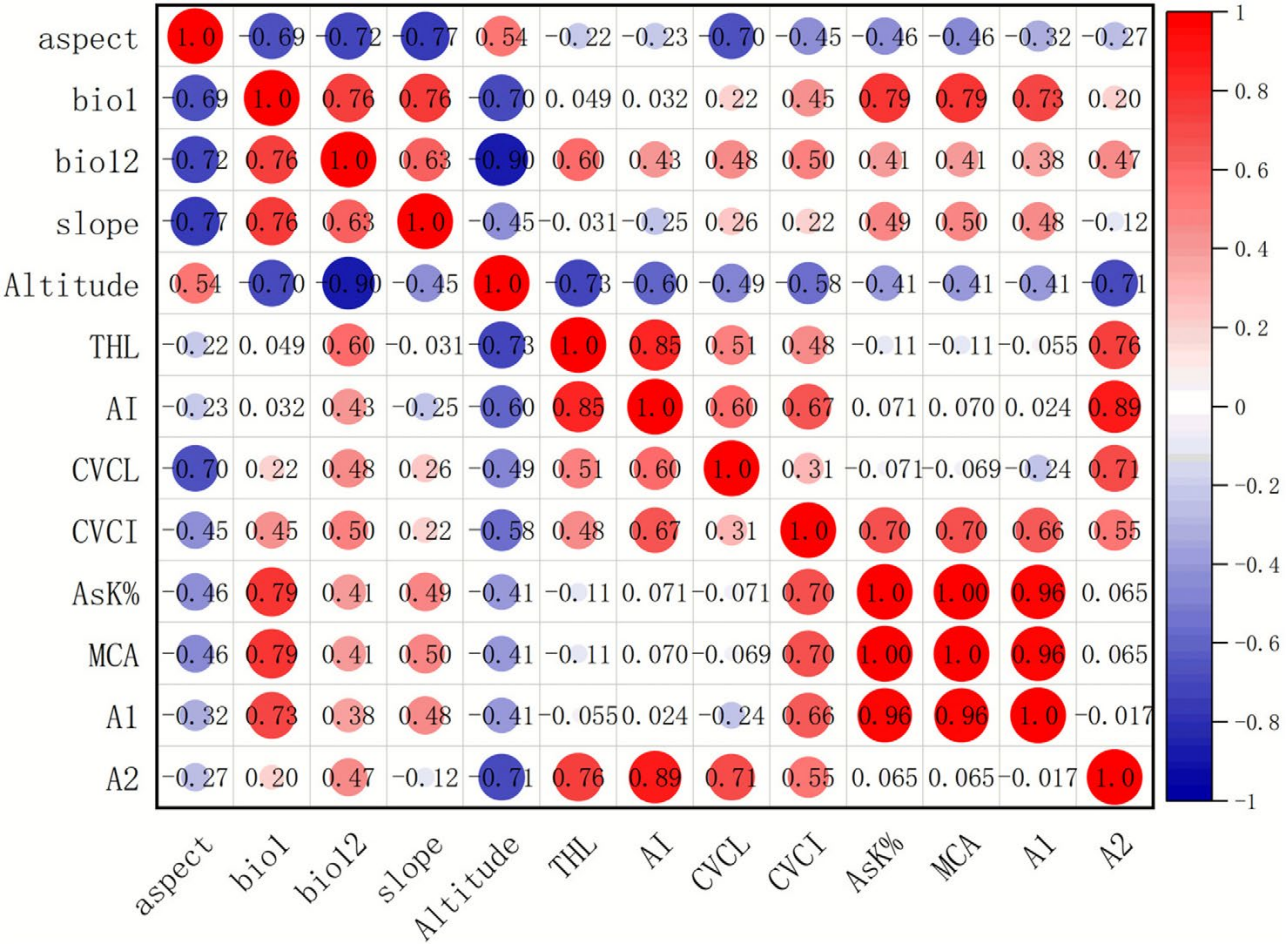
Correlation analysis between karyotypic parameters and major environmental factors.

**FIGURE 6:**
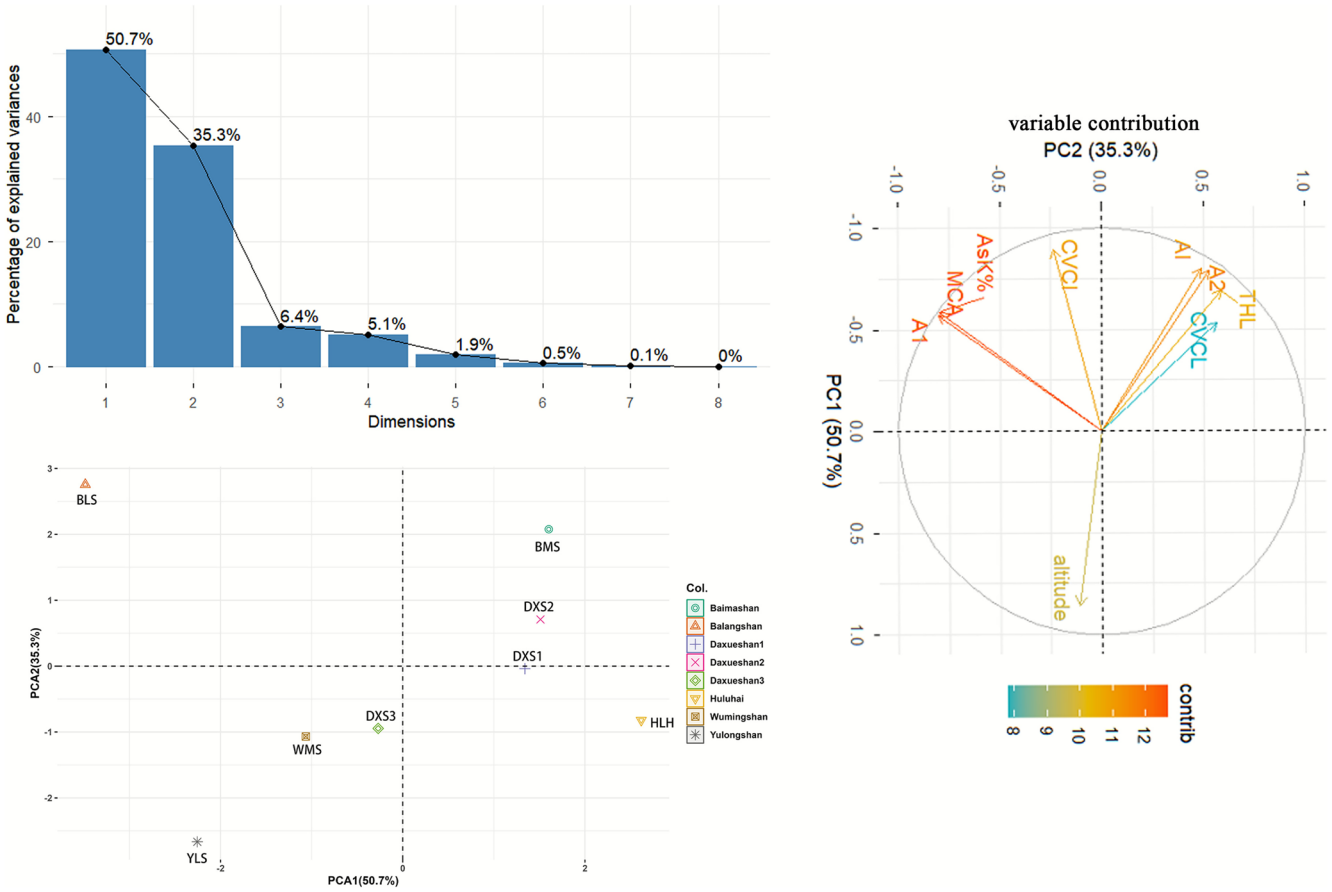
Principal component analysis (PCA) of karyotype parameters and altitude for eight *Lilium lophophorum* populations.

The eight *L. lophophorum* populations were named according to their collection sites: Daxueshan1 (DXS1), Daxueshan2 (DXS2), Daxueshan3 (DXS3), Yulongshan (YLS), Huluhai (HLH), Wumingshan (WMS), Baimashan (BMS), and Balangshan (BLS) (Table 1). Analysis of the eight populations yielded one principal finding, supported by cytological and statistical evidence:

1. Key Finding: Significant chromosomal variation was observed among populations, including differences in morphology and karyotype
2. Cytological Evidence: Chromosomal features, karyotype images, and detailed data for each population are shown in Figure 3, Table S1, Table 2, and Figure 4 (mitotic cytograms of *L. nanum* ).
3. Statistical Evidence: Statistical analyses using karyotype indices, Pearson correlation coefficients (Figure 5), and PCA (Figure 6) further demonstrate chromosomal diversity across populations, with additional data provided in Table S2.

**TABLE 2:** Cytological parameters of eight *Lilium lophophorum* populations.

Population	Chromosome number\base number\ploidy	THL	AI	CVCL	CVCI	AsK%	MCA	A1	A2	Karyotype	Karyotype formula
DXS1	24\12\2	136.06	7.43	18.11	48.08	78.76	57.52	0.73	0.18	3A	2*n* = 2*x* = 24 = 18st + 2t + 2sm + 2 m
DXS2	24\12\2	150.42	7.77	17.35	44.8	78.46	56.96	0.73	0.17	3A	2*n* = 2*x* = 24 = 16st + 2t + 4sm + 2 m
DXS3	24\12\2	148.9	9.27	13.12	49.78	79.2	58.39	0.74	0.19	3A	2*n* = 2*x* = 24 = 18st + 2t + 2sm + 2m
YLS	24\12\2	140.43	8.28	19.07	51.83	80.28	60.55	0.75	0.19	3A	2*n* = 2*x* = 24 = 16st + 4t + 2sm + 2m
HLH	24\12\2	132.52	7.13	15.45	46.15	78.78	57.55	0.73	0.15	3A	2*n* = 2*x* = 24 = 18st + 2t + 2sm + 2m
WMS	24\12\2	141.76	10	18.82	53.15	79.56	59.11	0.74	0.19	3A	2*n* = 2*x* = 24 = 16st + 4t + 2sm + 2m
BMS	24\12\2	139.35	8.86	20.24	43.76	78.26	56.54	0.72	0.2	3B	2*n* = 2*x* = 24 = 16st + 4t + 2sm + 2m
BLS	24\12\2	175.82	12.47	23.22	53.71	78.80	57.60	0.73	0.23	3B	2*n* = 2*x* = 24 = 16st + 4 t + 4 m

### 3.1 Chromosomal Characteristics and Karyotype Classification

*L. lophophorum* chromatin displayed two distinct interphase patterns: dense dispersion (e.g., DXS2 and HLH) or complex chromocenters (e.g., BMS). Mitotic metaphase counts showed 24 chromosomes in all eight *L. lophophorum* populations, with a basic number of 12 and no variation in chromosome number or ploidy. The first and second chromosome pairs were long with central or subcentral centromeres. Chromosome length distributions varied among populations: DXS1 (9.31–16.24 μm), DXS2 (10.39–17.88 μm), DXS3 (10.31–18.46 μm), YLS (9.20–16.38 μm), HLH (9.43–15.63 μm), WMS (9.63–17.47 μm), BMS (8.19–16.69 μm), and BLS (22.85–10.33 μm). No satellite chromosomes were observed.

Secondary constrictions occurred on the long arms of chromosome pairs 3 and 4 in DXS1, HLH, DXS2, WMS, and BMS; on pairs 3, 4, 5, and 6 in YLS; and on pairs 2, 3, 4, and 5 in DXS3. The karyotype asymmetry index (Ask%) ranged from 78.26 to 80.28, with YLS showing the highest value. The coefficient of variation of the centromeric index (CVCI) ranged from 43.76 to 53.71, and the coefficient of variation of chromosome length (CVCL) ranged from 13.12 to 23.22. According to Stebbins' classification, all populations exhibited a 3A karyotype type except BMS and BLS, which were 3B. DXS1, HLH, and DXS3 shared the same karyotype formula (2*n* = 2*x* = 24 = 18st + 2t + 2sm + 2m), while YLS, WMS, and BMS shared another (2*n* = 2*x* = 24 = 16st + 4t + 2sm + 2m). Both differed from DXS2 (2*n* = 2*x* = 24 = 16st + 2t + 4sm + 2 m) and BLS (2*n* = 2*x* = 24 = 16st + 4t + 4m).

### 3.2 Correlations Between Altitude and Karyotype Parameters

Correlation heatmap analysis (Figure 5) revealed a complex set of relationships between karyotype parameters and environmental factors. Altitude showed a negative correlation with several key karyotypic indices (including total haploid length [THL], Ask%, A1, and A2), with THL decreasing at higher elevations. This pattern suggests that *L. lophophorum* may exhibit genomic reduction as a potential response to high‐altitude environments, although the underlying mechanisms remain unclear. Maybe a smaller, perhaps more tightly packed genome is more resistant to UV‐induced damage (Norsang et al. 2011; Mao et al. 2021; Wang et al. 2021). This result is consistent with some studies (Xu et al. 2024), but contrasts with others (Guo et al. 2018; Feng et al. 2022) reporting increased genome size with elevation. Given the limited research on altitude‐genome size relationships, these correlations remain inconclusive and may reflect lineage‐specific adaptive strategies on the QTP.

PCA (Figure 6) further highlighted the influence of environmental factors, particularly altitude, on karyotypic variation. The first two principal components (PC1 and PC2) collectively explained 86% of total variance, with PC1 accounting for 50.7%. Population distribution in the PCA scatterplot indicated clear karyotypic differentiation in YLS and BLS relative to the other populations, potentially reflecting their lower‐altitude preferences.

Autocorrelation analysis was not conducted due to limited population‐level sampling, which would preclude reliable assessment. Moreover, the goal of this analysis was to explore preliminary associations between karyotypic parameters and environmental factors rather than to construct predictive models. Thus, the results should be considered exploratory. We propose that altitude may influence karyotypic differentiation in *L. lophophorum*, but confirmation will require larger sample sizes and more comprehensive analyses.

### 3.3 Species Distribution Modeling

**FIGURE 7:**
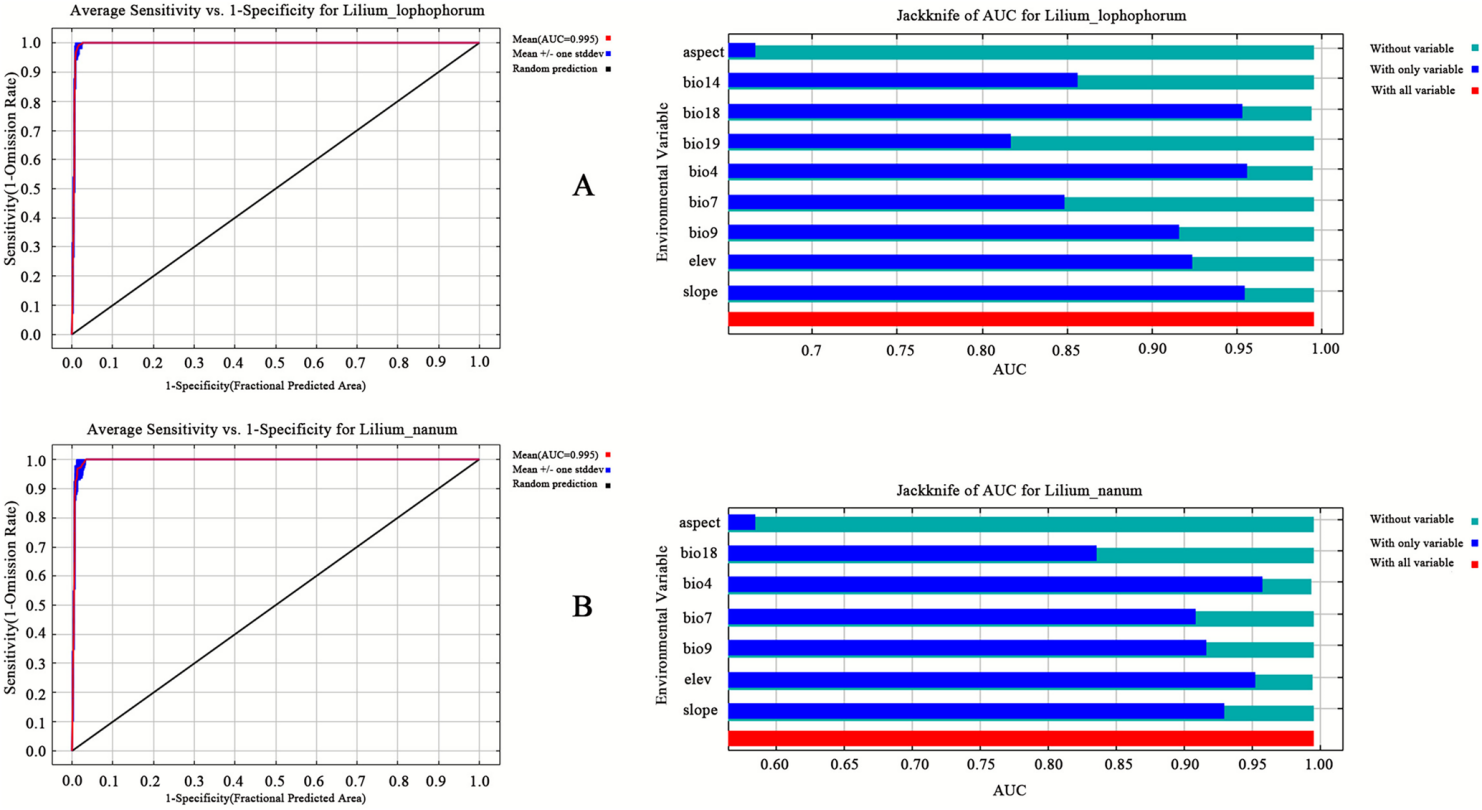
Receiver operating characteristic (ROC) curves and Jackknife area under the curve (AUC) analyses for *Lilium lophophorum* and *Lilium nanum* based on environmental variables. (A) ROC curve (left) and Jackknife analysis (right) for *L. lophophorum*, with an AUC of 0.995 indicating excellent model performance and showing the contribution of each environmental variable. (B) ROC curve (left) and Jackknife analysis (right) for *L. nanum* , also with an AUC of 0.995, confirming strong model performance and variable contributions.

**FIGURE 8:**
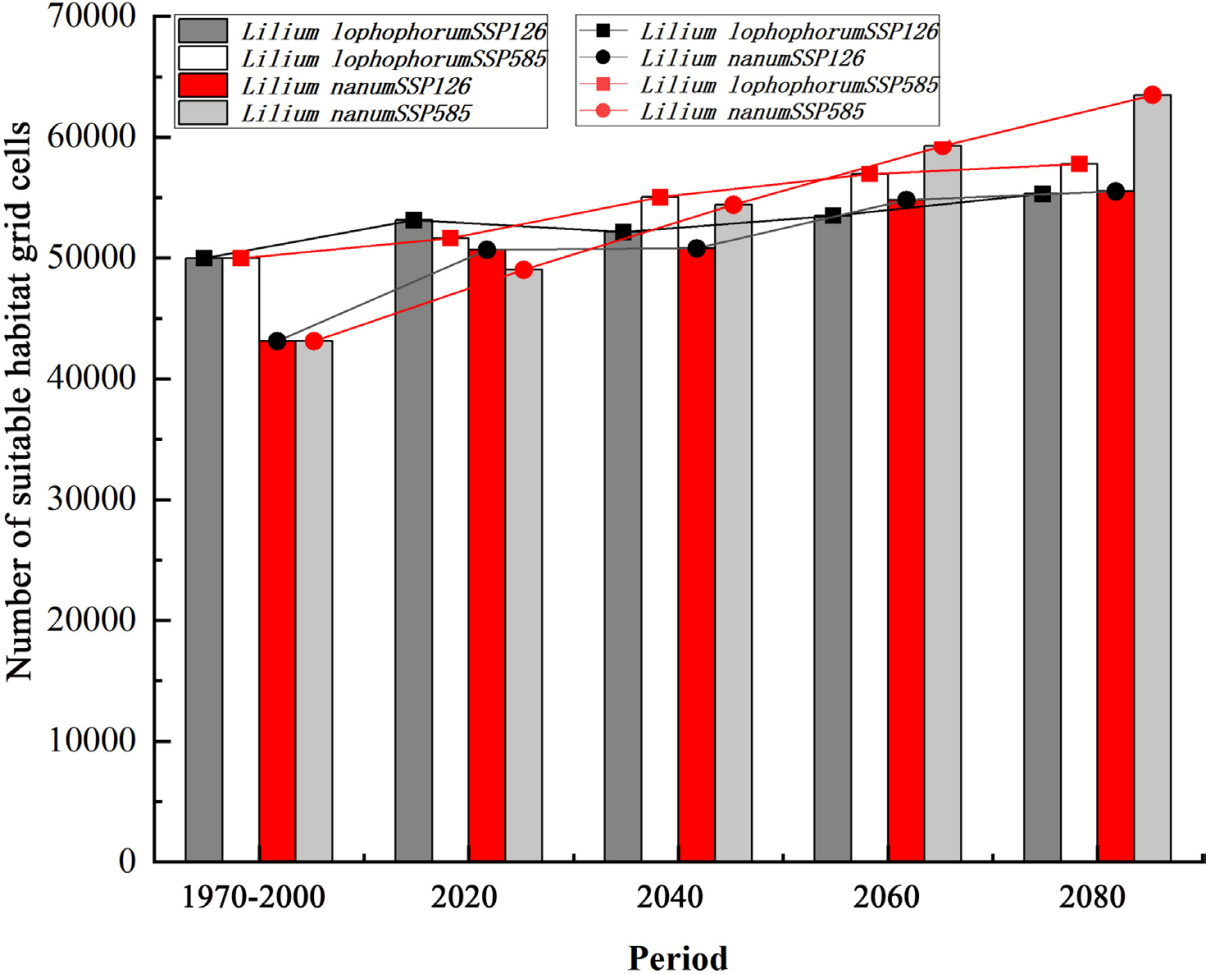
Predicted changes in suitable habitat areas of *Lilium lophophorum* and *Lilium nanum* under SSP126 and SSP585 climate scenarios from 2000 to 2080. The y‐axis shows the number of suitable habitat grid cells, and the x‐axis spans the present to 2080 (x‐axis). SSP126 represents a low‐emission scenario and SSP585 represents a high‐emission scenario.

**FIGURE 9:**
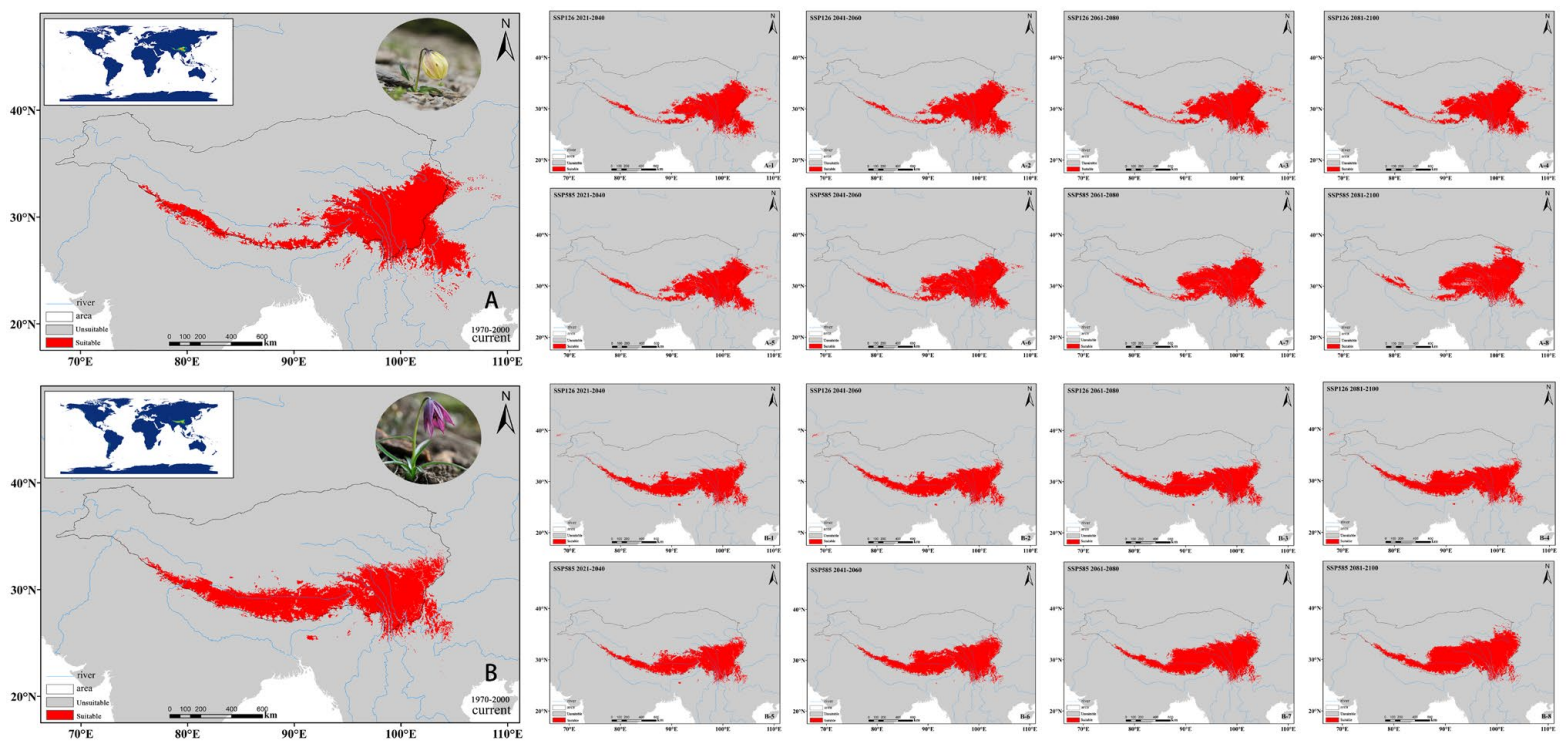
Current and projected habitat suitability for *Lilium lophophorum* (A) and *Lilium nanum* (B) under SSP126 and SSP585 climate scenarios from 2020 to 2080. Habitat suitability is shown in red (suitable) or gray (unsuitable).

The modeled distributions of the two *Lilium* species in the Tibetan Plateau and Hengduan Mountains showed high accuracy (AUC = 0.995) (Figure 7). Jackknife analysis identified bio4 (temperature seasonality), bio9 (mean temperature of the driest quarter), elev (elevation), and slope as the most influential variables for both species (AUC ≥ 0.9) (Figure 7). Model results indicated that suitable habitats for both *L. nanum* and *L. lophophorum* expand under both the SSP126 and SSP585 climate change scenarios, with a more pronounced increase under SSP585. The expansion was greater for *L. nanum* , suggesting that emissions‐driven warming may be driving both species toward higher latitudes for suitable conditions (Table 3, Figures 8 and 9).

**TABLE 3:** Projected range sizes of *Lilium lophophorum* and *Lilium nanum* under SSP126 and SSP585 climate scenarios (1970–2080).

Taxon	Range size*								
	1970–2000	2020		2040		2060		2080	
		SSP126	SSP585	SSP126	SSP585	SSP126	SSP585	SSP126	SSP585
*Lilium lophophorum*	49,987	53,138 (+)	51,652 (+)	52,150 (+)	55,035 (+)	53,5 02 (+)	56,954 (+)	55,310 (+)	57,795 (+)
*Lilium nanum*	43,107	50,670 (+)	49,012 (+)	50,804 (+)	54,394 (+)	54,798 (+)	59,263 (+)	55,513 (+)	63,478 (+)

* Range size refers to the change in suitable habitat area, measured as the number of grid cells. “+” indicates an increase in area.

## 4 Discussion

Previous karyotype studies in *Lilium* have shown generally stable chromosome numbers, with most Liliaceae species being diploid (2*n* = 24). Exceptions such as *Cardiocrinum giganteum* (2*n* = 2*x* = 22) (Zhu et al. 2002) and the triploid *Lilium lancifolium* (2*n* = 3*x* = 36) (Yang et al. 1996) demonstrate that variation in chromosome number and ploidy, though uncommon, does occur within the genus.

Our study confirms that *L. lophophorum* is diploid (2*n* = 24) with no B chromosomes, consistent with earlier reports (Li and Chen 1985), but reveals a previously underappreciated degree of intraspecific karyotypic diversity. Wild *Lilium* species typically exhibit bimodal karyotypes, with two pairs of long chromosomes bearing subcentral (sm) or central (m) centromeres and ten pairs of short chromosomes with subterminal (st) or terminal (t) centromeres, usually classified as Stebbins' type 3B. While most populations in our study shared this general organization, notable deviations were observed: most populations exhibited a 3A karyotype, whereas only the BMS and BLS conformed to the typical 3B type reported for wild *Lilium* species from northeast China and for *Lilium davidii*.

According to Stebbins' view that “symmetric is primitive, asymmetric is evolutionary,” the high karyotype asymmetry indices (78.26%–80.28%) observed across populations suggest that *L. lophophorum* represents a primitive yet actively evolving group. The evolutionary ranking of the eight populations, from highest to lowest asymmetry, was: YLS ≥ WMS ≥ DYS3 ≥ BLS ≥ HLH ≥ DYS1 ≥ DYS2 ≥ BMS. Notably, the population with the highest asymmetry (YLS) occurred at the lower elevation, implying that lower‐altitude environments may be associated with faster evolutionary change in this species.

Additional evidence of intraspecific diversification appears in the distribution of secondary constrictions. These were mainly located on the long arms of pairs 3 and 4 across populations, with extra constrictions on pairs 5 and 6 in YLS. Because secondary constrictions are associated with structural modifications such as translocations and inversions, their presence and variation indicate ongoing chromosomal evolution. CVCL also varied among populations, with BLS showing the highest value (23.22). Given that chromosomal structural changes are often linked to heterogeneous habitats and broad distribution ranges, environmental factors, particularly those that shift with altitude such as temperature and humidity, are likely major drivers of the karyotypic diversity observed in *Lilium*.

Using the MaxEnt model, we predicted changes in suitable habitats for the two *Lilium* species under alternative climate change scenarios. The results indicate a general shift toward higher latitudes and a significant expansion of suitable habitat at higher elevations, particularly under SSP585. The projected upward shift was more pronounced in tetraploid *L. nanum* than in diploid *L. lophophorum*. Although our data do not establish causation, the association between polyploidy and a stronger upward range expansion suggests that polyploidization may enhance the capacity to occupy newly suitable high‐altitude habitats.

China's diverse and widely distributed *Lilium* species constitute an important genetic resource for breeding and diversity research. Alpine meadow populations, in particular, provide valuable germplasm for developing cold‐adapted varieties. Cytological data from karyotype and niche analyses offer insight into karyotypic variation that can inform targeted breeding strategies. MaxEnt projections of future habitat shifts underscore the need for proactive conservation, with high‐altitude regions, as critical germplasm reservoirs, warranting priority protection.

## 5 Conclusions

Based on an integrated cytogenetic and ecological analysis of two endemic alpine lilies, this study establishes a descriptive foundation for their chromosomal diversity and distribution patterns. It documents substantial intraspecific karyotypic variation in the diploid *L. lophophorum* (2*n* = 24), with preliminary associations to altitude, and presents the first chromosomal characterization of the tetraploid *L. nanum* (2*n* = 48). Ecological modeling under future climate scenarios predicts upward range shifts for both species, with the tetraploid showing greater potential for habitat expansion. The observed distribution patterns, together with the dwarf phenotype of *L. nanum* , support the hypothesis that polyploidy may enhance resilience in high‐altitude environments, suggesting a possible adaptive advantage. However, given the limited sample size, these results should be viewed as hypothesis‐generating rather than demonstrating adaptive superiority. This work provides a theoretical framework for future research, emphasizing the need for expanded sampling and genomic analyses to test the link between polyploidy and environmental adaptability. The findings also contribute to broader understanding of alpine plant evolution and offer insights relevant to the conservation of high‐altitude refugia under climate change.

## Author Contributions

**Tengfei Gui:** conceptualization (equal), formal analysis (equal), methodology (equal), software (equal), writing – original draft (equal). **Mingyue Lin:** conceptualization (equal), writing – original draft (equal). **Zhiming Li:** supervision (equal). **Deli Peng:** data curation (equal). **Yuan Huang:** writing – review and editing (equal). **Wenguang Sun:** conceptualization (equal), data curation (equal), funding acquisition (equal), project administration (equal), writing – original draft (equal).

## Funding

Please add: This research was supported by the Second Tibetan Plateau Scientific Expedition and Research (STEP) program (2024QZKK0200), the Key Projects of the Joint Fund of the National Natural Science Foundation of China (U23A20149), the National Natural Science Foundation of China (32360065), the Key Research and Development Program of Yunnan Province (202403AC100028), National Key R&D Program of China (2024YFF1306700), the Yunnan Applied Basic Re‐search Project (202401AT070102, 202201AU070057).

## Conflicts of Interest

The authors declare no conflicts of interest.

## Supporting information


**Table S1:** Chromosomal parameters of eight *Lilium lophophorum* populations.
**Table S2:** Karyotypic and environmental factor analysis data.

## Data Availability

The data presented in this study are available in this article (see tables and figures); species distribution data were obtained from GBIF (https://www.gbif.org/) and iPlant (http://www.iplant.cn/), while environmental data came from WorldClim (https://www.worldclim.org/) and the Chinese Academy of Sciences (http://www.gscloud.cn/). The slope and aspect were derived from DEM data.
