# Supplementary material for: Karyotyping and Distribution Patterns of Endemic Chinese Lilies: Insights Into Their Conservation Under Climate Change
Source: Ecol Evol. 2026 Jan 11;16(1):e72824. doi: 10.1002/ece3.72824 (PMC12790874; doi:10.1002/ece3.72824)
Supplement: Supplementary file 1 — Table S1: Chromosomal parameters of eight Lilium lophophorum populations. Table S2: Karyotypic and environmental factor analysis data. [file ECE3-16-e72824-s001.docx]

Table S1. Chromosomal parameters of eight *Lilium lophophorum* populations

| **Population** |  | **Haploid Table** | | | **Arm ratio** | **Type** |
| --- | --- | --- | --- | --- | --- | --- |
|  |  | Long arms | Short arms | Chromosome length (μm) |  |  |
|  | 1 | 9.42 | 6.83 | 16.24 | 1.42 | m |
|  | 2 | 9.21 | 5.16 | 14.37 | 1.79 | sm |
|  | 3 | 10 | 1.73 | 11.73 | 5.82 | st |
|  | 4 | 10 | 1.48 | 11.47 | 6.8 | st |
|  | 5 | 10.03 | 1.33 | 11.36 | 7.62 | t |
|  | 6 | 9.29 | 1.71 | 11 | 5.45 | st |
| DXS 1 | 7 | 9.17 | 1.51 | 10.69 | 6.09 | st |
|  | 8 | 8.9 | 1.77 | 10.67 | 5.02 | st |
|  | 9 | 8.72 | 1.37 | 10.09 | 6.37 | st |
|  | 10 | 7.26 | 2.42 | 9.68 | 3.01 | st |
|  | 11 | 7.74 | 1.72 | 9.46 | 4.48 | st |
|  | 12 | 7.43 | 1.88 | 9.31 | 4.01 | st |
|  | 1 | 10.4 | 7.48 | 17.88 | 1.4 | m |
|  | 2 | 10.35 | 5.4 | 15.75 | 1.91 | sm |
|  | 3 | 11.39 | 1.6 | 12.98 | 7.19 | t |
|  | 4 | 10.78 | 1.66 | 12.44 | 6.51 | st |
|  | 5 | 10.6 | 1.78 | 12.38 | 5.99 | st |
| DXS 2 | 6 | 10.28 | 1.8 | 12.08 | 5.72 | st |
|  | 7 | 10.01 | 1.93 | 11.95 | 5.18 | st |
|  | 8 | 9.36 | 1.91 | 11.26 | 4.92 | st |
|  | 9 | 8.66 | 2.53 | 11.2 | 3.51 | st |
|  | 10 | 9.41 | 1.72 | 11.13 | 5.49 | st |
|  | 11 | 9.02 | 1.97 | 10.99 | 4.59 | st |
|  | 12 | 7.77 | 2.62 | 10.39 | 2.99 | sm |
|  | 1 | 10.9 | 7.56 | 18.46 | 1.44 | m |
|  | 2 | 9.77 | 5.75 | 15.52 | 1.71 | sm |
|  | 3 | 10.42 | 1.7 | 12.12 | 6.14 | st |
|  | 4 | 10.46 | 1.61 | 12.08 | 6.5 | st |
|  | 5 | 10.37 | 1.68 | 12.04 | 6.21 | st |
| DXS 3 | 6 | 10.39 | 1.54 | 11.93 | 6.74 | st |
|  | 7 | 10.12 | 1.74 | 11.85 | 5.87 | st |
|  | 8 | 10.4 | 1.43 | 11.83 | 7.3 | t |
|  | 9 | 9.35 | 1.92 | 11.27 | 4.89 | st |
|  | 10 | 9.22 | 1.69 | 10.91 | 5.48 | st |
|  | 11 | 8.1 | 2.5 | 10.6 | 3.27 | st |
|  | 12 | 8.44 | 1.88 | 10.31 | 4.5 | st |
|  | 1 | 9.71 | 6.67 | 16.38 | 1.46 | m |
|  | 2 | 8.81 | 4.69 | 13.51 | 1.88 | sm |
|  | 3 | 9.71 | 1.31 | 11.02 | 7.43 | t |
|  | 4 | 9.29 | 1.41 | 10.69 | 6.6 | st |
|  | 5 | 9.27 | 1.34 | 10.61 | 6.92 | st |
| YLS | 6 | 8.87 | 1.4 | 10.27 | 6.32 | st |
|  | 7 | 9.15 | 1.08 | 10.23 | 8.48 | t |
|  | 8 | 8.44 | 1.41 | 9.85 | 5.99 | st |
|  | 9 | 8.16 | 1.57 | 9.72 | 5.25 | st |
|  | 10 | 8.03 | 1.47 | 9.49 | 5.46 | st |
|  | 11 | 7.63 | 1.84 | 9.47 | 4.15 | st |
|  | 12 | 7.65 | 1.55 | 9.2 | 4.97 | st |
|  | 1 | 10.09 | 1.36 | 11.45 | 7.44 | t |
|  | 2 | 9.49 | 1.4 | 10.89 | 6.76 | st |
|  | 3 | 8.98 | 6.65 | 15.63 | 1.35 | m |
|  | 4 | 8.52 | 4.39 | 12.92 | 2.09 | sm |
|  | 5 | 7.74 | 2.44 | 10.18 | 3.21 | st |
| HLH | 6 | 9.4 | 1.51 | 10.91 | 6.23 | st |
|  | 7 | 8.95 | 1.52 | 10.48 | 5.88 | st |
|  | 8 | 9.07 | 1.63 | 10.7 | 5.57 | st |
|  | 9 | 8.27 | 1.62 | 9.89 | 5.13 | st |
|  | 10 | 8.13 | 1.66 | 9.79 | 4.91 | st |
|  | 11 | 7.68 | 1.76 | 9.43 | 4.37 | st |
|  | 12 | 8.09 | 2.18 | 10.27 | 3.76 | st |
|  | 1 | 10.23 | 1.29 | 11.52 | 7.95 | t |
|  | 2 | 9.72 | 1.32 | 11.04 | 7.35 | t |
|  | 3 | 10.17 | 1.48 | 11.65 | 6.86 | st |
|  | 4 | 10.16 | 1.56 | 11.72 | 6.51 | st |
|  | 5 | 9.12 | 1.44 | 10.56 | 6.36 | st |
| WMS | 6 | 9.74 | 1.64 | 11.38 | 5.97 | st |
|  | 7 | 8.99 | 1.65 | 10.65 | 5.45 | st |
|  | 8 | 9.38 | 1.86 | 11.24 | 5.06 | st |
|  | 9 | 7.88 | 1.75 | 9.63 | 4.52 | st |
|  | 10 | 7.84 | 2.12 | 9.96 | 3.77 | st |
|  | 11 | 9.92 | 7.55 | 17.47 | 1.31 | m |
|  | 12 | 9.65 | 5.33 | 14.98 | 1.81 | sm |
|  | 1 | 9.88 | 6.81 | 16.69 | 1.45 | m |
|  | 2 | 9.16 | 5.34 | 14.5 | 1.72 | sm |
|  | 3 | 11.29 | 1.56 | 12.85 | 7.23 | t |
|  | 4 | 10.92 | 1.46 | 12.37 | 7.51 | t |
|  | 5 | 10.2 | 2.08 | 12.29 | 4.94 | st |
| BMS | 6 | 9.9 | 1.6 | 11.51 | 6.18 | st |
|  | 7 | 8.9 | 2.45 | 11.36 | 3.64 | st |
|  | 8 | 8.64 | 1.83 | 10.48 | 4.73 | st |
|  | 9 | 8.1 | 1.87 | 9.97 | 4.37 | st |
|  | 10 | 7.82 | 1.93 | 9.75 | 4.06 | st |
|  | 11 | 7.49 | 1.92 | 9.41 | 3.91 | st |
|  | 12 | 6.76 | 1.43 | 8.19 | 4.77 | st |
|  | 1 | 13.02 | 9.83 | 22.85 | 1.33 | m |
|  | 2 | 11.32 | 7.17 | 18.48 | 1.59 | m |
|  | 3 | 14.61 | 1.64 | 16.25 | 9.01 | t |
|  | 4 | 13.94 | 1.86 | 15.79 | 7.56 | t |
|  | 5 | 12.67 | 2.21 | 14.87 | 5.74 | st |
| BLS | 6 | 12.26 | 1.83 | 14.09 | 6.89 | st |
|  | 7 | 11.73 | 1.71 | 13.44 | 6.92 | st |
|  | 8 | 11.19 | 1.98 | 13.17 | 5.74 | st |
|  | 9 | 10.65 | 2.13 | 12.78 | 5.02 | st |
|  | 10 | 9.94 | 2.13 | 12.06 | 4.78 | st |
|  | 11 | 9.41 | 2.30 | 11.71 | 4.21 | st |
|  | 12 | 7.82 | 2.51 | 10.33 | 3.38 | st |

Table S2: Karyotypic and environmental factor analysis data.

| **Species** | **longitude** | **latitude** | **bio1(℃)** | **bio12**  **(mm)** | **aspect** | **slope** | **altitude**  **(m)** | **THL** | **AI** | CVCL | CVCI | **AsK%** | MCA | A1 | A2 |
| --- | --- | --- | --- | --- | --- | --- | --- | --- | --- | --- | --- | --- | --- | --- | --- |
| Daxueshan1 | E99°50′21.13" | N28°35′3.83" | -0.002166656 | 666 | 255.9172058 | 11.36616993 | 4509.00 | 136.06 | 7.43 | 18.11 | 48.08 | 78.76 | 57.52 | 0.73 | 0.18 |
| Yulongshan | E100°10′45.68″ | N27°01′54.66" | 7.348332882 | 798 | 135.6255951 | 18.34535027 | 3924.00 | 140.43 | 8.28 | 19.07 | 51.83 | 80.28 | 60.55 | 0.75 | 0.19 |
| Huluhai | E99°57′45.691" | N28°31′16.802" | -0.35983339 | 688 | 241.1165009 | 8.47282505 | 4693.00 | 132.52 | 7.13 | 15.45 | 46.15 | 78.78 | 57.55 | 0.73 | 0.15 |
| Daxueshan2 | E99°88'97.98" | N28°58'44.76" | -0.111000001 | 696 | 246.9976959 | 11.67041016 | 4335.00 | 150.42 | 7.77 | 17.35 | 44.80 | 78.46 | 56.96 | 0.73 | 0.17 |
| Daxueshan3 | E99°83'57.23" | N28°58'57.26" | 0.268999994 | 681 | 351.555603 | 2.784214973 | 4280.00 | 148.90 | 9.27 | 13.12 | 49.78 | 79.20 | 58.39 | 0.74 | 0.19 |
| Wumingshan | E100°1'19.79" | N29°7'45.34" | -0.207833305 | 646 | 224.8016968 | 6.285517216 | 4525.00 | 141.76 | 10.00 | 18.82 | 53.15 | 79.56 | 59.11 | 0.74 | 0.19 |
| Baimashan | E99°1'17.68" | N28°23'15.63" | -0.493833303 | 659 | 268.2020874 | 3.440875053 | 4471.00 | 139.35 | 8.86 | 20.24 | 43.76 | 78.26 | 56.54 | 0.72 | 0.20 |
| Balangshan | E102°55'14.61" | N30°53'27.23" | 1.378332973 | 803 | 173.6287994 | 9.37731266 | 3852.00 | 175.82 | 12.47 | 23.22 | 53.71 | 78.80 | 57.60 | 0.73 | 0.23 |
